# Spontaneous juggling behavior in a wild urban capuchin monkey (*Sapajus nigritus cucullatus*)

**DOI:** 10.1007/s10329-026-01265-0

**Published:** 2026-05-27

**Authors:** Gabriel Leite Saraiva, Guilherme Akira Awane, Rafaela Guglak Cavichia, Julia dos Santos Gutierres, David Lins Fernandes Leiroza Lovato, Lucas M. Aguiar, Ana Paula Vidotto-Magnoni

**Affiliations:** 1 Laboratório de Ecologia e Comportamento Animal, Departamento de Biologia Animal e Vegetal, Rodovia Celso Garcia Cid, PR 445 Km 380, Campus Universitário, Universidade Estadual de Londrina, Londrina, Paraná Brazil; 2 Laboratório de Símios, Departamento de Zoologia, Programa de Pós-graduação em Zoologia, Universidade Federal do Paraná (UFPR), Coronel Francisco H. dos Santos Av., 100, Curitiba, Paraná 81531- 980 Brazil

**Keywords:** Behavioral innovation, Emergent behavior, Motor coordination, Object play, Urban ecology, Urban wildlife

## Abstract

Juggling, defined as the repeated and rhythmic throwing and catching of an object, is a rare and complex form of object manipulation among non-human animals. While object play has been described in several taxa, true juggling behavior, intentional, successive aerial manipulation of the same object, has only been formally documented in a few species such as otters, dolphins, corvids, and, more recently, in gibbons and macaques. In this study, we report the first known case of juggling behavior in a wild free-ranging Platyrrhini, a juvenile black-horned capuchin monkey (*Sapajus nigritus cucullatus*) inhabiting an urban forest fragment in southern Brazil, with access to human foods. The event was recorded opportunistically during a behavioral study of object manipulations by capuchins. The juvenile, positioned alone on the ground in a green open area, picked up a coconut and performed a sequence of seven successive throws and recoveries, each executed with precision, rhythm, and apparent intentionality. This observation expands the known taxonomic and ecological range of juggling-like behaviors and underscores the cognitive and motor flexibility of *Sapajus* species. It also raises questions about the influence of urban environments on behavioral innovation in wild primates, where exotic objects are present and predation and competition may be more relaxed, freeing up time for innovations. We propose that this object manipulation represents an innovative play behavior in a juvenile capuchin, which, beyond serving as a ludic activity, may function as a means of cognitive engagement, motor practice, and possibly cultural transmission.

**Supplementary Information:**

The online version contains supplementary material available at 10.1007/s10329-026-01265-0.

## Introduction

Juggling is a rare and complex form of object manipulation that involves the repeated throwing, catching, or coordinated movement of objects, oftenly associated with skillful entertainment or performance in human culture, but in animals, juggling-like behaviors, defined as rhythmic, non-functional tossing and recapturing of objects, are usually interpreted as a form of play (Fagen 1981). Reports of juggling behavior in wild animals are extremely scarce and primarily limited to a few taxa (Deng and Zhou 2016). These behaviors require advanced motor coordination, attention, and often a degree of cognitive engagement, making them particularly interesting from an ethological and evolutionary perspective (Bekoff and Byers 1998; Pellis and Pellis 2009).

One of the earliest evidence of juggling-like behavior (what may be described as *proto-juggling*) reported in scientific literature was observed in corvids during the 1990s. Heinrich and Smolker (1998) reported that juvenile ravens (*Corvus corax*) engaged in object play that included the aerial tossing, dropping, and retrieval of twigs and stones, often in a solitary context. Although “juggling” was not applied at the time, its key features include: repeated, rhythmic object manipulation and apparent intentionality. In the early 2000s, similar behavior was registered in captive Asian small-clawed otters (*Aonyx cinereus*), which were seen tossing pebbles between their paws, often while lying on their backs (Allison et al. 2020).

The first scientific use of the term *“*juggling*”* in a primate context occurred in a study of wild Hainan gibbons (*Nomascus hainanus*), in which adult males were seen repeatedly throwing and catching sticks while moving through the canopy (Deng and Zhou 2016). These actions were consistent across individuals and interpreted as deliberate play or motor display. In long-tailed macaques (*Macaca fascicularis*), stone-handling behaviors include a wide variety of manipulations, some of which involve tossing and retrieving stones (Pelletier et al. 2017).

Capuchin monkeys (*Cebus* spp. and *Sapajus* spp.) exhibit great behavioral plasticity and can learn and modify their repertoire, including object-related behaviors, according to different environmental influences (Ottoni and Izar 2008; Falótico and Ottoni 2023; Bender and Aguiar 2025). Also, playful manipulation often precedes and scaffolds functional tool use (Falótico and Ottoni 2013). In wild populations, capuchins engage in diverse forms of play with objects, with studies in Brazil documenting not only playful object use, but also the spontaneous invention of tool-like behaviors, such as using sticks to probe holes (Ottoni and Izar 2008). Juvenile capuchins are particularly active in exploratory and playful behaviors, which may include manipulating fruits, stones, sticks, or other environmental elements (Fragaszy et al. 2004; Bender and Aguiar 2025). Moreover, immature capuchins have a high tendency to produce behavioral innovations (Goldsborough et al. 2025). These findings underscore the cognitive flexibility of *Sapajus* and support the idea that play behavior—particularly with objects—may serve as a platform for behavioral innovation in the genus.

In urban environments, the ecological conditions, such as abundant human objects and food, low predation pressure and reduced interspecific competition, could increase free time and foster behavioral innovations (Gavriilidi et al. 2022). Here, we describe an unusual case of object juggling behavior exhibited by a free-ranging juvenile black-horned capuchin monkey (*Sapajus nigritus cucullatus*), inhabiting an urban forest fragment in southern Brazil.

## Methods

**Fig. 1:**
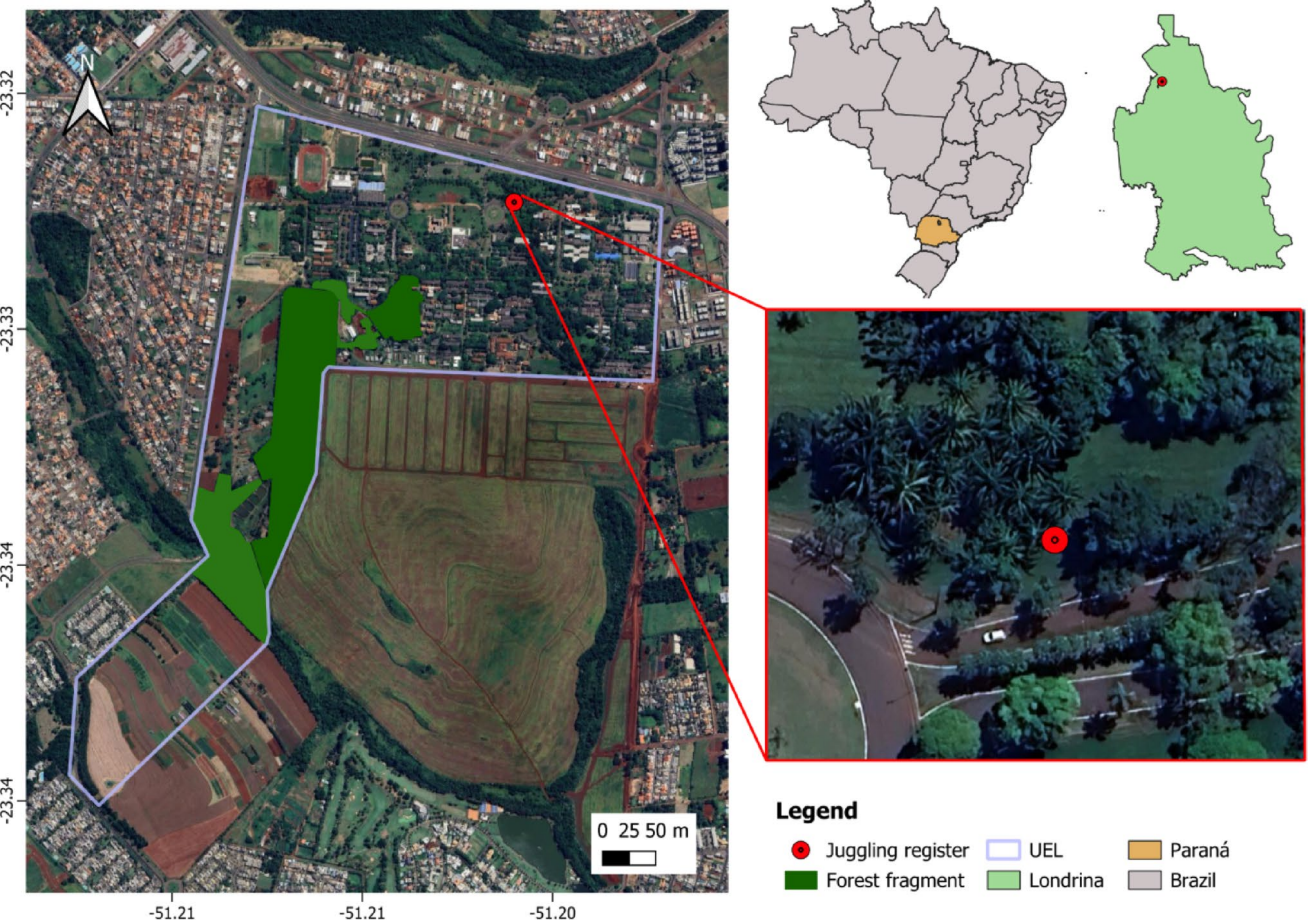
Location of the juggling behavior displayed by a black-horned capuchin monkey recorded at the UEL site, Londrina, Paraná, Southern Brazil (23°19’25.31"S, 51°11’57.82"W)

This study was conducted on the campus of State University of Londrina (hereafter: UEL), an urban forested environment located in the municipality of Londrina, Paraná, southern Brazil (23°19’25.31"S, 51°11’57.82"W) (Fig. 1). The area is embedded within an urban matrix and is characterized by several fragments of secondary Seasonally Semideciduous Forest, a subtype of the Atlantic Forest (Rezende et al. 2018). The study was authorized by IBAMA/ICMBio (authorization number 84428-1), by the Ethics Committee on Animal Use of UEL (CEUA - n⁰ 007.2023) and followed the standard practices for field primatology (MacKinnon et al. 2014).

The UEL site (23°19′31.09″S, 51°11′59.92″W) covers 235 hectares and comprises a mosaic of paved areas, buildings, agricultural areas and forest patches (Fig. 1). The largest fragment is the *Horto Florestal*, with approximately 20 hectares. The black-horned capuchin monkeys (*Sapajus nigritus cucullatus*) at UEL live in a free-ranging regime and are highly habituated to human presence (Pereira et al. 2026). They frequently use both natural and human-modified environments, moving on paved paths and accessing trash cans around the campus, where they frequently manipulate exotic objects and consume high-caloric human-made foods. Their forest habitat is depauperated and natural competitors and predators are scarce or absent, although they can be threatened by dogs and domestic cats (Pereira et al. 2026). Our research team has been monitoring these primates since 2015.

**Fig. 2:**
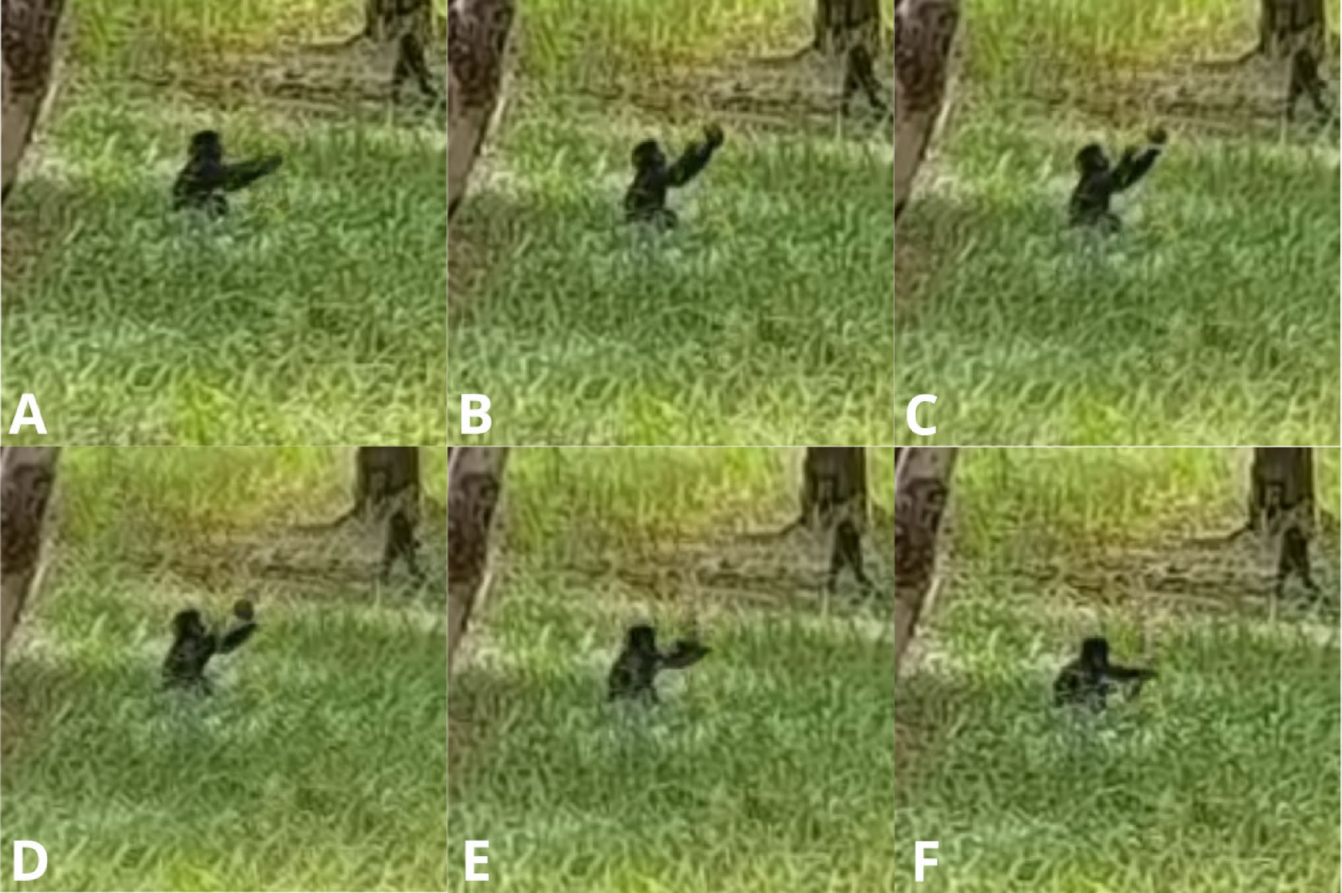
Sequential movements (from left to right and top to bottom) of juggling object-throwing behavior by a juvenile black-horned capuchin monkey (*Sapajus nigritus cucullatus*) observed at the study site

With the aim to study object manipulations by these capuchins, a single group was followed by the first author from July 2023 to June 2024. The area was visited for two weeks each month, with three days of fieldwork per week, totaling approximately 25 h of direct animal observation and data collection. During the study, the group size ranged from 37 individuals (3 adult males, 16 adult females, and 18 juveniles) at the beginning to 34 individuals by the end of the sampling period. Object manipulations were recorded through focal animal sampling (Altmann 1974), with 5 min of sampling every 5 min interval for each visible individual, but the juggling behavior was registered during the interval between focal samplings with the aid of a smartphone camera and was described *ad libitum*. The video recording was reviewed and processed using the free version of DaVinci Resolve (Blackmagic Design). The original video was separated into 354 individual frames, that were inspected sequentially to examine the behavioral sequence in detail. Representative frames illustrating the successive stages of the object manipulation were selected to compose the sequential image presented in Fig. 2.

## Results

The juggling behavior was observed in a juvenile individual on January 19th, 2024, at 14:48. The juvenile was foraging on the ground in a green open area of the UEL campus (Fig. 2), at a point about two meters away from where it found a dried coconut. The juvenile picked it up and sat alone on the ground, positioned between two other juveniles from its group, each located 2–4 m away. At one point, the juvenile threw the coconut upward and the object rotated in the air before falling back into the monkey’s hands (Fig. 2), after which it was thrown again six more times in succession. The entire episode lasted approximately 11 s (Supplementary Material 1). After the episode, the monkey abandoned the coconut and moved to another location in the ground where it rested and foraged.

During the activity, the individual maintained a slightly hunched posture, manipulating the object in a coordinated manner. The action consisted of successive upward throws, using the hands with precision and rhythm. With each toss, the monkey visually tracked the coconut’s trajectory, demonstrating attentiveness and readiness to catch it mid-air or, if it fell to the ground, to retrieve it and resume the cycle. To execute each throw, the monkey used both arms and kept both hands open to recapture the object with ordinary grips. The throws and recoveries occurred in rapid succession, indicating motor fluidity and continuous engagement.

The animal’s body movements followed the object’s dynamics, with subtle postural adjustments that facilitated its retrieval. The control over the force and direction of the throws indicates a remarkable degree of motor coordination, concentration, and intentionality. The behavior appeared to be deliberate, with no apparent association with feeding, defense, social activity or practical use of the object. This episode was recorded during a single day of contact with the group and was not observed again during subsequent data collection.

## Discussion

The observed juggling behavior by a juvenile *S. n. cucullatus* represents a novel and remarkable addition to the repertoire of object manipulation and solitary play in wild Platyrrhini. Although object manipulation, including throwing, is relatively common in capuchins (Fragaszy et al. 2004), the structured, rhythmic repetition of tossing and recapturing a single object—as documented in this case—has not yet been reported in individuals of the genus. The behavior appears to meet key criteria for what has been described in other taxa as “juggling”: intentional, repeated throwing and catching of an object in a non-utilitarian, play-like context (Deng and Zhou 2016; Allison et al. 2020). A similar behavior was observed by Perry & Manson (2008) in *Cebus capucinus*, in which a male manipulated a *Sloanea terniflora* fruit as if it was juggling it instead of trying to eat it. The observation was unique and lacks detail, but because these fruits have stinging hairs, they cannot be manipulated easily, in part explaining why this was the only observation (O’Malley and Fedigan 2005; Perry and Manson, 2008).

This instance expands the taxonomic and ecological scope of juggling-like behaviors, previously recorded in species with high motor dexterity and cognitive complexity, such as otters (Allison et al. 2020), dolphins (Mackey et al. 2014), corvids (Heinrich and Smolker 1998), as well in primates, including long-tailed macaques (Pelletier et al. 2017) and Hainan gibbons (Deng and Zhou 2016), which inhabit sanctuary temples and natural reserves, respectively. In most of these species, juggling has been interpreted as a form of object play possibly related to skill development, cognitive stimulation, or social signaling.

The current observation in *S. n. cucullatus* aligns with these interpretations mentioned above, particularly given the apparent deliberateness, attentional engagement, and motor coordination displayed by the juvenile. Also, the hand movements observed in the episode follows the pattern of object grasping observed in the genus, consisting of holding an object in the palm of the hand with partially flexed fingers and the parallel alignment of the thumb with the other fingers, in the so called ‘power grip’ (Napier 1956; Truppa et al. 2019).

The fact that this behavior was displayed by a solitary juvenile, exhibited a repetitive motor pattern, and occurred in a non-feeding context without any associated agonistic or manipulative goals supports the hypothesis that it constitutes a form of play (Bekoff and Byers 1998). Play behavior, especially object play, is often considered a mechanism for motor refinement and exploratory learning (Pellis and Pellis 2009). In primates, it has also been proposed as a precursor to functional tool use, particularly when it involves exploratory manipulation of objects (Fragaszy and Perry 2003). Capuchins are known for their manual dexterity and natural propensity for tool use in some populations (Ottoni and Izar 2008). Although *S. n. cucullatus* has not been widely documented using tools in the wild (Gutierres et al. 2025), its cognitive flexibility and sensorimotor capabilities may still support the emergence of complex play and tool using routines like the one described here, in both natural and anthropized areas (Resende and Ottoni 2002; Kerney et al. 2017; Jordan et al. 2021).

Urban environments may provide a unique context for the emergence or expression of behavioral innovations in capuchin monkeys (Aguiar et al. 2014; Nina e Silva et al. 2017). The abundance of caloric foods, reduced guild competition and reduction of natural predators, as is the case of the UEL site, can release free time for capuchin innovations (Gavriilidi et al. 2022). Thus, these monkeys can be living with more free time when compared with other populations living in more pristine habitats. Increased exposure to anthropogenic objects, novel substrates, and spatial variability may also enhance opportunities for exploratory and play behaviors in primates, since innovation emerges from already known behaviors used in new contexts (Reader and Laland 2001). The juggling behavior observed in the forested environment located within an urban matrix suggests that such modified environments could serve as incubators for behavioral novelty. This possibility raises new questions regarding how urbanization may influence the behavioral repertoire of wild primates, including the expression of playful or symbolic behaviors rarely observed in less disturbed habitats.

The brevity and rarity of the event recorded in this group restricts broader generalizations, but its atypicality is intriguing and the motor pattern is so reminiscent of human juggling, that its description is worthy in itself (Thierry 2023), and its cataloguing is important to generate new hypotheses (Bicca-Marques 2003). Besides, it remains unclear whether this behavior is an individual innovation, a transient exploratory action, or part of an emerging or yet undocumented cultural tradition within this group.

Future longitudinal observations may help determine the frequency, social transmission, and developmental trajectory of juggling-like behaviors in wild capuchins. They may also help in understanding the motivational and functional roles of these behaviors, including their potential links to motor skill development, cognitive engagement, and socio-environmental context. Given the apparent novelty of such behaviors in urban-dwelling populations, research should also explore how anthropogenic environments influence the emergence, expression, and possibly the transmission of playful innovations like this one. Such efforts will be essential to broadening our understanding of the diversity, ecological drivers, and evolutionary significance of object play in primates.

This record of juggling behavior in *S. n. cucullatus* underscores the behavioral richness of Platyrrhini and highlights the potential for spontaneous ludic innovation even in very disturbed environments or, indeed, stimulated by them. It also invites further exploration into how play, cognition, and motor skill development intersect in wild primate populations.

## Supplementary Information

Below is the link to the electronic supplementary material.

Supplementary Material 1
